# Personalized Reimbursement Model (PRM) program: A real-world data platform of cancer drugs use to improve and personalize drug pricing and reimbursement in France

**DOI:** 10.1371/journal.pone.0267242

**Published:** 2022-04-19

**Authors:** Pierre-Alexandre Squara, Vinh-Phuc Luu, David Pérol, Bruno Coudert, Valérie Machuron, Camille Bachot, Laurence Samelson, Virginie Florentin, Jean-Marc Pinguet, Béchir Ben Hadj Yahia

**Affiliations:** 1 Department of Medicine, University of Paris, Paris, France; 2 Medical Affairs Department, Roche, Boulogne-Billancourt, France; 3 Medical Oncology Department, Centre Léon Bérard Comprehensive Cancer Center, Lyon, France; 4 Medical Oncology Department, Georges Francois Leclerc Comprehensive Cancer Center, Dijon, France; 5 Medical Evidence Department, Roche, Boulogne-Billancourt, France; 6 Market Access Department, Roche, Boulogne-Billancourt, France; 7 Personalized Healthcare Department, Roche, Boulogne-Billancourt, France; Chung Shan Medical University, TAIWAN

## Abstract

**Objective:**

This article describes the Personalized Reimbursement Model (PRM) program methodology, limitations, achievement and perspectives in using real-world data of cancer drugs use to improve and personalize drug pricing and reimbursement in France.

**Materials and methods:**

PRM platform aggregates Electronic Pharmacy Records (EPR) data from French medical centers (PRM centers) to build retrospective cohorts of patients treated with injectable cancer drugs in a hospital setting. Data extracted on January 1^st^, 2020, from breast cancer (BC) patients who received trastuzumab, trastuzumab emtansin or pertuzumab since January 1st, 2011, and from lung cancer (LC) patients who received bevacizumab or atezolizumab since January 1^st^, 2015, enabled recovering their injectable cancer drugs history from diagnosis date until December 30^th^, 2019, and served as dataset for assessment.

**Results:**

123 PRM centers provided data from 30,730 patients (25,660 BC and 5,070 LC patients respectively). Overall, 20,942 (82%) of BC and 4,716 (93%) of LC patients were analyzed. Completion rate was above 98% for patients characteristics, diagnostic and treatment related data. PRM centers cover 48% and 33% of BC and LC patients in-hospital therapeutic management in France, respectively. Distribution of BC and LC patients therapeutic management, by medical center category and geographic location, was similar in PRM centers to all French medical centers, ensuring the representativeness of the PRM platform.

**Conclusion:**

PRM Platform enabled building a national database generating on demand Real-World Evidence based on EPR. This enabled the first performance-based risk-sharing arrangements based on PRM data, between the CEPS and Roche, for atezolizumab cancer immunotherapy in metastatic non-small cell lung cancer indication.

## Introduction

These last decades, the number of new cancer cases has been rising annually in Europe, and reached 382,000 in France in 2018, mainly related to population growth and its ageing [1, 2]. In the meantime, cancer mortality rates decreased due to improved access to earlier diagnosis, as well as successive waves of therapeutic innovations [2]. While these innovations improve survival rates and quality of life for many patients, their high prices brings new challenges for healthcare system sustainability, especially in terms of appropriate access and budget impact [3, 4]. French National Health Insurance spending on innovative and costly in-hospital cancer drugs (T2A list drugs) have risen by 52% since 2011, reaching €2.2bn in 2018 [5]. Between 2015 and 2018, only 27 (63%) of the 43 new cancer drugs who received an EU marketing authorization were fully available to French patients, 8 (19%) were not available and 8 (19%) had limited availability [6]. In the same period, the average time between the cancer drug EU marketing authorization and access for patient was 579 days (excluding early access programs) ranking France 19^th^ out of 35 EU countries in terms of time to drug availability [6]. Therefore, improving access and regulating budget impact of therapeutic innovations in oncology has become a joint concern for health authorities, patient associations and pharmaceutical companies.

The French pricing and reimbursement (P&R) model, a key factor in drug access and budgetary impact, has not sufficiently adapted to the rapid evolution of therapeutic innovation in oncology [7]. Currently, P&R decisions for innovative and costly in-hospital cancer drugs in France are made by health authorities (after the drug has been granted a Marketing Authorization and before the product is launched on market) and mainly depends on medical evidence from randomized clinical trials (RCT) [8]. Although RCT remain the best available standard, increasing number of cancer drugs face challenges to align with the traditional drug development pathway, especially personalized or rare cancer drugs, where only preliminary evidence from promising albeit not validated surrogate end-points or interim analyses may be available, or for which RCT are unfeasible or unethical [9]. Furthermore, clinical trials are conducted under strict adherence to structured protocols and restrictive eligibility criteria partially reflecting routine clinical practices [10, 11]. Consequently, uncertainties remain regarding the real-world use, effectiveness or cost-effectiveness of cancer drugs when assessed by health authorities. These situations could lead to limited or delayed patient access to innovation. Furthermore, a cancer drug has an undifferentiated cost per vial regardless its indication, including when used in combination with other cancer drugs. This is particularly true for immune checkpoint inhibitors that can be used in multiple indications to treat different cancers, as monotherapy or in combination with other cancer drugs and where a number of patients will not benefit from the treatment due to primary or adaptive resistance [12]. Drugs cost should be driven by the value it delivers to the patient and therefore differentiated for each indication and population based on its effectiveness to avoid resources misallocation.

An option to improve cancer drugs access and budget impact lies in further incorporating the use of real-world evidence (RWE) in drugs P&R models by: 1/ promoting early access programs (ATU) conditional on real-world data (RWD) collection of safety and effectiveness, especially for patients with limited treatment options, 2/ encouraging full access to innovative cancer drugs approved by EMA and adjusting their access and reimbursement based on timeliness real-world effectiveness, 3/ adjusting drugs price according to their use in real word or their effectiveness to optimize resource allocation [13, 14].

To date, RWD have played a minor role in the P&R process in France, mainly because their implementation is hindered by: 1/ hurdles to efficiently capture health data (data availability and quality), 2/ restrictive data sharing policy, 3/ lack of sustainability in data collection and analysis, and 4/ difficulties to generate robust RWE [15, 16]. Having an appropriate data infrastructure able to capturing good quality and representative data in a sustainable manner, and generate promptly on demand robust RWE is an essential requirement to increase the use of RWD at each step of the health technology assessment.

In contrast to most available databases in France, PRM platform is designed to process on demand electronic pharmacy records (EPRs) data informing payers decisions, and can provide results in a few weeks with sufficient coverage and representativeness to produce robust and scalable RWE. Electronic Pharmacy Records (EPRs) are widely implemented and used in French medical centers for over twenty years by physicians and pharmacists in their routine practice for prescription, dispensation and administration of injectable cancer drugs. EPRs contain patient characteristics, diagnostic and treatment related data. Initiated in 2014, the PRM program aims at aggregating EPR data of breast and lung cancers patients treated in French medical centers.

This article describes the Personalized Reimbursement Model (PRM) program methodology, limitation, achievement and perspective in using real-world data of cancer drugs use to improve and personalize drug pricing and reimbursement in France.

## Materials and methods

### Description of PRM platform

PRM platform aggregates EPR data from 130 public and non-public French medical centers involved in the PRM program (PRM centers) in a centralized database (PRM database). Each data extraction from PRM database enables building retrospective cohorts of patients who received at least one injection of a cancer drugs of interest (trastuzumab, trastuzumab emtansin, pertuzumab, bevacizumab or atezolizumab), and recovering their injectable cancer drugs history (drugs on the T2A-list and outside the T2A-list) from diagnosis date until data extraction date. Extracted variables mainly include patient characteristics, diagnostic and treatment related data. All variables extracted are presented in Table 1. PRM centers extract, pseudonymize and deposit EPR data on PRM platform provided by a third-party ISO-27001 certified hosting provider Advanced Schema. In accordance to the authorization given by the French Data Protection Authority (CNIL), only the Roche data processor, Advanced Schema, is described as the recipient of the dataset. The Information Notice given to the patients does not provide a specific information about sharing the data with other third parties. In this context, the sharing of the pseudonymized dataset is not permitted. However, the dataset can be shared for legal obligations with authorities. PRM program is compliant with European and French legislation regarding health data, data protection and patient information. This work was supported by Roche. Roche was involved in the statistical analysis.

**Table 1:** List of data extracted from electronic pharmacy record systems and data completion rates in the breast cancer and lung cancer analyzable population.

		Data completion rates	
Type of information	Entry name	Breast cancer analyzable population	Lung cancer analyzable population
Center data	Center ID number	100.0%	100.0%
Patient characteristics	Hospital patient ID number	100.0%	100.0%
	Birth year	99.9%	100.0%
	Gender	99.9%	100.0%
	Weight	99.9%	99.6%
	Height	99.9%	99.6%
	ECOG Performance status	NA	11,7%
Diagnostic data	Diagnosis	99.9%	99.2%
	Disease stage	100.0%	100.0%
	Locations of metastases	< 5%	7,6%
	Oncogenic drivers	31.1% (HER2)	8–12%
			(ALK, EGFR, PD-L1)
Treatment data	Drug name	100.0%	100.0%
	Drug quantity	100.0%	100.0%
	Administration date	100.0%	100.0%
	Treatment line	100.0%	100.0%
	Cycle number	98.5%	98.7%
	Frequency	90.9%	98.7%
	Treatment regimen	99.9%	99.5%
	Estimated duration	99.9%	99.5%
Treatment response	Results of disease assessment	7.0%	< 5%
	Disease assessment date	< 5%	< 5%
Treatment discontinuation	Discontinuation motive	< 5%	< 5%
	Discontinuation date	< 5%	< 5%
Involvement in clinical trials	Clinical trial name	98.0%	99.0%

BRAF, B-RAF proto-oncogene; ECOG, Eastern Cooperative Oncology Group; EGFR, Epidermal Growth Factor Receptor; HER2, Human Epidermal growth factor Receptor 2; INN, International Nonproprietary Name; KRAS, Kirsten RAt Sarcoma viral oncogene homolog; NA, Not Applicable, PD-1, Programmed cell Death-1 receptor; PD-L1, Programmed cell Death-1 Ligand. Cycle number: incremental number of each cycle; Frequency: duration between each cycle (in days); Estimated duration: estimated duration of each treatment regimen; Results of disease management: complete response, partial response, disease progression, stable disease; Treatment regimen: dosing and timing of drugs administration and frequency.

### Data management

The first step of data management consists in codifying and standardizing unstructured free text data extracted from different EPR systems (S1 Table). Data are then pooled and stored on a dedicated and secure database server.

The second step of data management consist in removing patients with: 1/ treatment sequences having unknown start date, i.e. the start of the treatment sequence is truncated, most likely due to an EPR software change, which does not allow to retrieve complete cancer drugs history, 2/ inconsistent cancer drugs name, i.e. brand name that does not match the International Nonproprietary Name (INN), 3/ inconsistent drug administration, i.e. two administrations of the same injected cancer drug on the same day, leading to the suspicion of a coding error, 4/ two or more concomitant primary cancer diagnoses reported, and 5/ administration of cancer drugs leading to the suspicion of the treatment of another concomitant cancer. A list of cancer drugs specific to the treatment of cancers other than BC or LC (S2 Table) has been validated and is regularly updated by an independent scientific committee involving oncologists, pharmacists and statisticians (S3 Table). Cancer drugs that are used in current practice to treat BC or LC, including cancer drugs used off-label or outside guidelines, are not on this list and are therefore captured by the PRM platform.

The third step of data management consists in applying algorithms to complete or correct data relative to disease stages and treatment lines when they are initially incomplete or missing. These algorithms consist in: 1/ retrieving relevant information from different entries in the database, 2/ checking information consistency over time and 3/ extracting relevant features to complete or correct data. These algorithms are detailed in S4 Table and their relevance has been supervised by the independent scientific committee. Patients with still missing, incomplete or inconsistent disease stage were excluded to reach a database with a population ready for analysis (i.e. PRM analyzable population).

### Assessment of completeness, coverage rate and representativeness

The potential of PRM program to capture real-world data of cancer drugs use to help inform drug pricing and reimbursement decisions in France, is assessed through three criteria: data completeness, coverage rate and representativeness of PRM database. As an example in this article, data extracted on January 1^st^, 2020 enabled building cohorts from BC patients who received trastuzumab, trastuzumab emtansin or pertuzumab between January 1^st^, 2011 and December 31^st^, 2019, and LC patients who received bevacizumab or atezolizumab between January 1^st^, 2015 and December 31^st^, 2019.

Completeness is measured by the proportion of available data for each variable in the PRM analyzable population (i.e. completion rate), after applying data management algorithms for disease stage and treatment line variables. Quality controls are performed to check that data management algorithms are correctly applied. For each algorithm, a patient sample of approximately 60 patients is selected and reviewed on the user interface (S1 Fig) presenting patient treatment related data, disease stage and treatment line before and after the application of data management algorithm to validate the correct application of the rule. For variables with a missing data rate higher than 30% (completeness below 70%) no imputation technique are performed and the variables are not considered for analysis.

Coverage rate at national level is controlled by comparing the numbers of BC and LC patients treated in PRM centers to those treated in all French medical centers through the French National Hospital database (PMSI). We assumed a target coverage rate of 33% of French BC and LC patients treated in PRM centers to be sufficient.

To ensure representativeness of BC and LC patients therapeutic management at national level, PRM centers were selected through a randomized stratified sampling method on medical center category and geographic location (region). This methodology should ensure that the patient characteristics in the PRM database are comparable to those in the national population.

The four medical center categories are: 1/ Comprehensive Cancer Centers (CCC), 2/ University Hospitals (UH), 3/ General Hospital (GH) and Non for-profit hospitals (NFPH), 4/ Private Hospital (PH). GH and NFPH were grouped based on similar practices and patient profiles. While more data are needed to describe in a more comprehensive way the practices and patient profiles in the different types of medical centers in France, a pragmatic approach based on healthcare professionals’ feedback was assumed. The six geographic locations are: 1/ Northern region, 2/ Eastern region, 3/ South-Eastern region, 4/ South-Western region, 5/ Western region and 6/ Paris area (S2 Fig). After each extraction, representativeness is controlled by comparing the distributions of BC and LC patients, by medical center category and geographic location, in PRM centers to all French medical centers, through PMSI.

## Results

### Population

**Fig 1:**
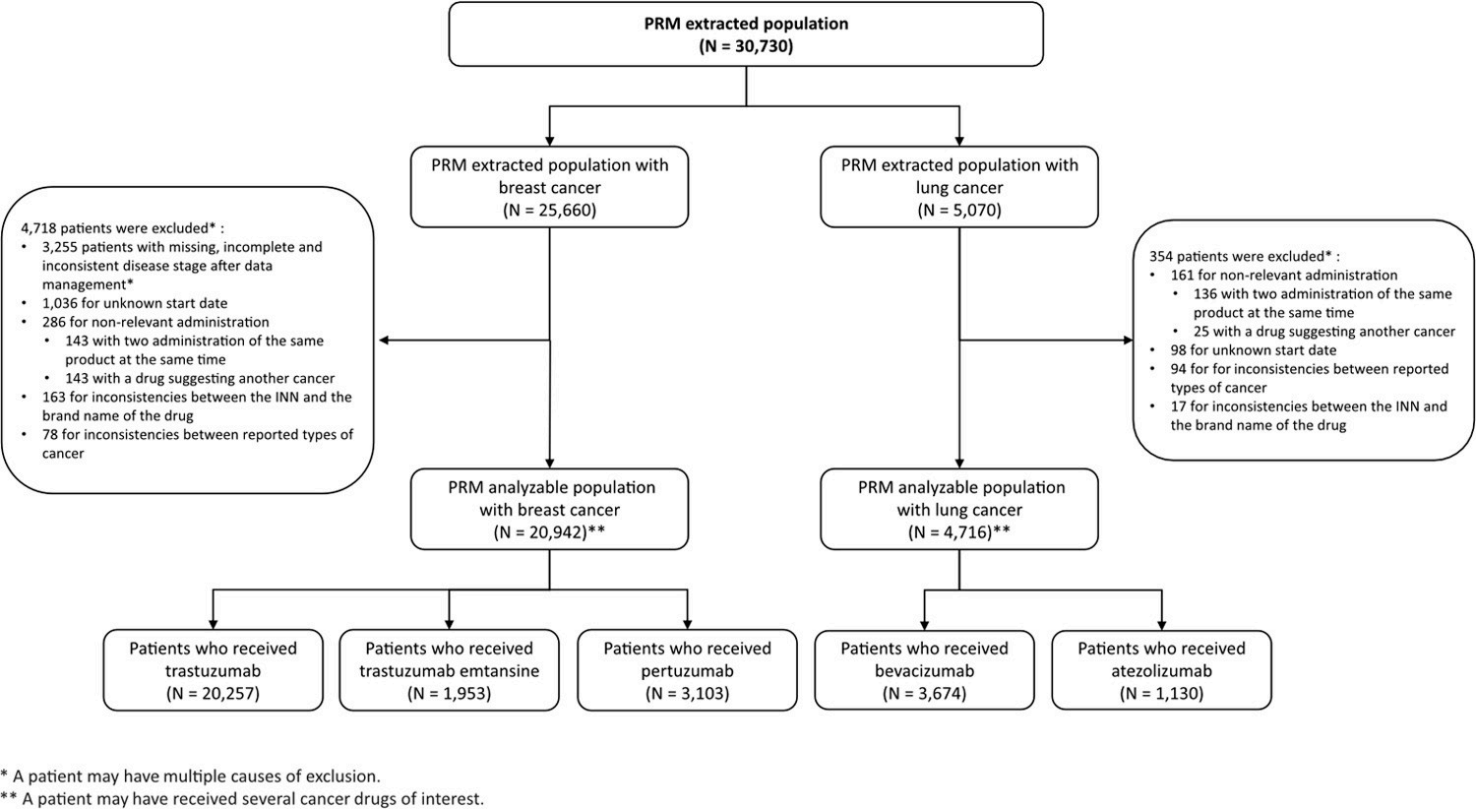
Flow chart. The flow chart represents the different steps of data management leading to the removal of patients from PRM extracted population, populated with data extracted by the French medical centers participating in the PRM program, to PRM analyzable population which allows building retrospective cohorts of breast cancer and lung cancer patients who received at least one injection of a cancer drugs of interest (trastuzumab, trastuzumab emtansin, pertuzumab, bevacizumab or atezolizumab).

As of January 1^st^, 2020, among 130 PRM centers, 7 (5%) medical centers did not perform data transfer. Hence, 123 PRM centers provided data from 30,730 patients (25,660 BC patients and 5,070 LC patients). Then, 5,072 (16,5%) patients were excluded, mainly due to missing, incomplete or inconsistent disease stage after data management (3,255/5,072), as well as those with unknown start date of treatment sequences (1,134/5,072). PRM analyzable population included 25,658 patients (20,942 BC and 4,716 LC patients). Overall, 82% (20,942/25,660) of BC and 93% (4,716/5,070) of LC patients data from the PRM extracted population were included in the PRM analyzable population. The different steps of patient selection are described in the flow chart (Fig 1).

### Completeness, coverage rate and representativeness

Completion rates were above 98% for patient characteristics, diagnosis, and treatment related variables. Before the application of the data management algorithms, disease stages had completion rates of 93% and 77% and treatment lines had completion rates of 80% and 70% for BC and LC patients respectively. Data management algorithms completed or corrected 7% of BC and 24% of LC patients disease stages and 33% of BC and 43% of LC patients treatment lines, allowing a 100% completion rate for disease stages and treatment lines after application of the data management algorithms. Each of the 11 and 13 data management algorithms applied on BC and LC patients respectively, have been correctly applied for more than 94% of the reviewed patients. Details of the quality control results for each data management algorithms are presented in S5 Table. Variables related to metastatic location, oncogenic drivers, treatment response and treatment discontinuation had completion rates ranged from 3% to 30%. For each variable, completion rates are detailed in Table 1.

PRM centers covers 48% and 33% of BC and LC patients in-hospital therapeutic management in France, respectively. The coverage rate by medical center categories and geographic area are detailed in Table 2 and S6 Table. Distribution of BC and LC patients therapeutic management, by medical center category and geographic area, was similar in PRM centers to all French medical centers.

**Table 2:** Distribution of patients who were treated for breast cancer and lung cancer, by category of medical center, in PRM centers and in all French medical centers in 2018, according to the French National Hospital database (PMSI).

BREAST CANCER					
	**Patients in PRM centers**		**Patients across the country**		
Category of medical centers	**N**	**% in PRM**	**N**	**% in France**	**Coverage Rate**
**General hospitals and non-for-profit hospitals**	7,058	26.2%	17,784	29.2%	40%
**University hospitals**	1,420	5.3%	5,757	9.4%	25%
**Comprehensive cancer centers**	8,793	32.6%	17,372	28.5%	51%
**Private hospitals**	9,712	36.0%	20,036	32.9%	48%
Total	**26,983**	**100.0%**	**60,949**	**100.0%**	**48%**
LUNG CANCER					
	**Patients in PRM centers**		**Patients across the country**		
Category of medical centers	**N**	**% in PRM**	**N**	**% in France**	**Coverage Rate**
**General hospitals and non-for-profit hospitals**	8,406	49%	23,441	46%	36%
**University hospitals**	2,553	15%	11,193	22%	23%
**Comprehensive cancer centers**	1,933	11%	5,090	10%	38%
**Private hospitals**	4,129	24%	11,184	22%	37%
Total	**17,021**	**100%**	**50,908**	**100%**	**33%**

Patients in PRM centers: Patients treated in PRM centers until end of 2019

Patients across the country: Patient treated across the country in 2018 (extracted from French National Hospital database)

Discrepancies are observed for UHs and the South-Eastern region. In PRM centers, 5.3% of BC and 15% of LC patients are treated in UHs versus 9.4% and 22% at national level, respectively. In PRM centers, 7% of the BC and 5% of the LC patients are treated in the South-Eastern region versus 13% and 14% at national level, respectively. Distributions of BC and LC patients therapeutic management, by medical center category and geographic location, in PRM centers and in all French medical centers are detailed in S6 Table.

## Discussion

As of January 1^st^, 2020, PRM platform enabled capturing RWD from EPR systems among 123 public and non-public French medical centers covering more than a third of BC and LC patients in-hospital therapeutic management in France. PRM database is populated with 30,730 BC or LC patients EPRs data, filled in by physicians in their routine practice for cancer drug prescription, and reviewed by pharmacists for cancer drug dispensation, which enables having a high completeness and correctness for patients characteristics, diagnostic and treatment related data. Moreover, effective data management algorithms improve data quality for disease stage and treatment line. Patient in-hospital injectable cancer drugs history is linked to patient characteristics, diagnostic, disease stage and treatment line, over time. This enabled the computation of RWE of trastuzumab, trastuzumab emtansin, pertuzumab, bevacizumab or atezolizumab use, based on: 1/ target population and patient profiles that are likely to be treated in real-world setting, 2/ off-label use and compliance with guidelines, 3/ treatment indication, especially for label extension and combinations with other treatments, 4/ involvement in clinical trials or in early access programs (ATU) and 5/ benefit of the drug based on proxy indicators such as the number of drug injections or treatment duration. Equitable distribution of BC and LC patients treated in PRM centers, by medical center category and geographic location, ensures the representativeness of the RWE at national level. Data extractions have been honed for several years and are increasingly automated avoiding time lag between data capture and their availability for analyses. Furthermore, PRM data extractions are also available on demand, providing up-to date RWE for timeliness health care decision making across the product life cycle. Data collection requires neither additional workload by healthcare professionals nor clinical monitors hiring, thus ensuring the sustainability of data capture within the PRM program.

### Limitations

Patient characteristics, diagnostic, and treatment related variable have high data completeness and correctness since they are mandatory for cancer drug prescription and double-checked by physicians and pharmacists in their routine practice through the EPR software, except for disease stage and treatment line that are provided at physicians discretion. Data management algorithms were developed to infer these variables from available data in PRM database. Data management processes and algorithms are controlled and continually improved in order to adapt to disease therapeutic management updates, supervised by the independent scientific committee. However this may still be insufficient and a validation with medical charts or other data sources would be of interest. Comparison with other RWD sources would enrich our understanding of the PRM program validity and representativeness. We attempted to compare the patient characteristics of PRM cohorts with other patients cohorts or registries. Nevertheless, it seems that this is to date barely feasible. For methodologically valid comparison, PRM database should be compared to 1/ a database which allows to retrieve patient cancer drugs history at a national level, 2/ ensuring representativeness of BC and LC patients therapeutic management by medical center category and geographic location, 3/ for a similar population as PRM (patient having received at least one Roche treatment) and 4/ covering a period consistent with the January 2020 PRM extraction. To our knowledge such a patient’s cohort or registry is not available in France. A comparison with national medico-administrative databases might be considered, even if the possibility of isolating comparable patients cohorts remains limited by the difficulty of identifying, beyond a precise diagnosis, disease stage, treatment lines and their therapeutic indications, all the more so as treatments outside the T2A-list are not available. Such a study could nevertheless be an added value for the program and should be considered in a near future.

Other variables such as metastasis’s location, oncogenic drivers, treatment response and treatment discontinuation, are poorly completed by physicians because their collection is not mandatory in EPRs systems, while available in patient medical records. These variables are not currently used to generate RWE. Close collaborations are established with medical centers involved in PRM program to improve data quality through specific improvement actions and incentivization. For each extraction, a personalized feedback is provided to PRM centers through reports and dashboards to monitor their activities. Aggregated data at regional and national levels are also shared for comparison purposes and as incentives to improve data quality.

PRM platform aggregates EPRs data and enables recovering patients injectable cancer drugs history from diagnosis date until data extraction date. Nevertheless, in some situations, this drug history may suffer some gaps. One situation is the use of oral cancer drugs and non-hospital-based injected drugs that are rarely recorded in the EPRs since not mandatory to be delivered to patients. This causes missing treatment sequences, sometimes difficult to identify. EPR software are gradually evolving to capture oral treatments. Still, healthcare professionals need to compel themselves to complete EPR with oral treatments and non-hospital-based injected drugs. Another situation that may lead to missing treatment sequences is related to patient change from medical center. The lack of unique patient identifier does not allow to link EPRs data across medical centers. Even though, this has limited impact since cancer patients are assumed to rarely change from medical center during their cancer treatment in France, applications are being developed for EPR software to ensure continuity of patient follow-up across centers, including home hospitalization.

Representativeness and coverage rate may vary for each extraction depending on the involvement of medical centers in PRM program. However, PRM medical centers remain relatively committed and centers that leave the PRM program are systematically replaced. A lower involvement of medical centers in the South-Eastern region as well as a limited number of UHs may also affect the representativeness. Since costly innovative in-hospital cancer drugs are fully reimbursed by the National Health Insurance ensuring equal access to these costly treatments across regions and type of medical centers, limited impact on representativeness is expected [17].

### Perspective

In recent years, the PRM program enabled building a platform aggregating up-to-date, good quality and representative RWD of in-hospital injected cancer drug use [18]. PRM program enabled setting the first Performance-Based Risk-Sharing Arrangement (PBRSA) based on PRM data between the French Healthcare Products Pricing Committee (CEPS) and Roche for the first indication of atezolizumab cancer immunotherapy in second line advanced Non-Small Cell Lung Cancer (NSCLC) in February 2019. This PBRSA is based on paybacks to the French National Health Insurance according to the proportion of patients not benefiting from atezolizumab, calculated through treatment duration in real-world setting. Among the five cancer drugs tracked in the PRM program, atezolizumab was the only drug that has benefited from a PBRSA. The CEPS advocates over the last decade that PBRSA is of weak interest because it did not contribute enough to the control of uncertainties regarding drug effectiveness that remain when assessed by health authorities. The objective of the PRM program is to provide on demand, robust real-world data to generate PBRSAs based and precise, co-constructed and shared indicators allowing CEPS to regain interest in this type of contract. A recent collaboration with Unicancer (French Hospital Federation bringing together the 18 French CCC), allowing to co-build the Oncology Data Hub which capitalizes on the complementary expertise and database of the ESME platform (the most important oncology RWD source in Europe) and the PRM platform, opens new perspectives.

Extending PRM Program to other drugs or to other types of cancer does not seem to present major difficulties. Implementation costs are reduced compared to traditional observational studies and workload is limited since PRM program aggregate EPR data that are provided by physicians and pharmacists, in their routine practice. Nevertheless, the extension of PRM program to other drugs or indications may require the involvement of more PRM centers to ensure acceptable representativeness and coverage rate by PRM database and the endorsement of the French Data Protection Agency (CNIL).

Further improvements would involve monitoring drug safety and effectiveness parameters [19]. Such developments require better capturing performance status, comorbidities, oncogenic drivers, treatment response, patient reported outcomes, adverse events or death related data. The most efficient option is certainly to enrich the PRM database with data already available in other French databases. France has national exhaustive medico-administrative databases, such as The French National Health Data System (SNDS), offering a long follow-up and little patient drop out, allowing particularly to follow the patient’s hospital journey (i.e. hospitalization for cancer relapse or complication), consumption of outpatient cancer drugs (i.e. oral cancer drugs) and the cause and date of death [20]. Other health database such as French Shared Medical Record (DMP), Communicating Cancer file (DCC) or cancer registries would allow to capture clinical, oncogenic driver and treatment response related outcomes. Enriching the PRM database with such data currently faces three main hurdles: 1/ The difficulty to merge PRM database with French medico-administrative databases, such as the SNDS, due to data privacy issues. Even though a unique patient identifier to centralize patient data is available in France, its permanent access is regulated by law and restricted to public institutions. Its use for research, on an ad hoc basis, is very limited, 2/ The lack of interoperability across health information systems. Some medical centers have up to 300 different information technology (IT) applications; and the lack of interoperability between software, as well as services and digital tools bring additional hurdles, 3/ The lack of data quality [21, 22]. The DMP lacks data completeness, since it is not routinely used in clinical practice. The DCC, a common record that facilitates Decision-making Multidisciplinary Team Meetings (RCP), is difficult to process into a structured database since its content is mainly populated with free text. Contexts where multiple sources of information of varying quality from multiple stakeholders need to be balanced to inform decision-making. However, this may change with the Health Data Hub initiative that aims at concentrating and linking existing health databases in France to facilitate their use [23]. Furthermore, Social Security Financing Act Project (PLFSS) for 2021 reports a public healthcare investment plan with commitment to invest €1.4 billion over the next three years to modernize digital healthcare tools and address interoperability issues between healthcare IT systems.

To date, there is still a low acceptability of RWE where the outcome of interest is effectiveness. Methodological challenges arise from the fundamental fact that RWD are not collected in a research perspective and therefore suffer from multiple biases and confounders [24, 25]. However, advanced strategies to adjust for confounding factors and the various biases that may occur are continuously improving, reducing the challenge of translating RWD into RWE [26–28].

Europe is fortunate having a rich healthcare data collection; and several public-private initiatives have been launched to encourage cooperation between industry, regulators, HTA agencies, and other stakeholders in exploring tools and methods for the use of RWD such as the Oncology Data Network (ODN) aiming to be a collaborative European data-sharing platform to inform cancer care, or the European Health Data Space [29–31].

PRM and similar programs offer new perspectives to build a European platform aggregating EPRs data, and timely capturing cancer drug use and treatment strategies. The effective use of routinely collected data would require countries to improve their capabilities not only to collect and link data generated by health care providers, payers or other stakeholders in health care systems, but also to set a strong framework to generate valuable RWE. Implementing such a platform requires building a data framework with a high level of standardization and interoperability in an environment fostering trust and confidence regarding data quality and data privacy [32]. Therefore, a collaboration is crucial between public and private stakeholders, as well as between countries willing to utilize RWE to inform their healthcare decisions.

## Conclusions

PRM Platform enabled building a national database generating on demand Real-World Evidence based on hospitals Electronics Pharmacy Records. This enabled the first performance-based risk-sharing arrangements based on PRM data, between the CEPS and Roche, for atezolizumab cancer immunotherapy in metastatic NSCLC indication. PRM program envisions expanding by collaborating with other healthcare system stakeholders in order to improve integration of RWE at all stages of the product life cycle, which could improve patient access to innovative cancer drugs while ensuring the health care system sustainability.

## Supporting information

S1 Fig PRM user interface. The user interface allows to show anonymized patient’s treatment history according to the treatment line and the disease stage. Patient ID, centre ID, age, gender, weight and height have been removed to ensure patient anonymization. This example shows the contribution of the data management algorithmto recover treatment lines and disease stage that were initially incomplete or missing, based on patient’s available data in the database. The top graphic presents the patient cancer drug history: the international nonproprietary name (INN) of the cancer drugs administered, the dose in mg and the date of administration for each cancer drugs administered. The graphic in the middle presents disease stage evolution and periods when the patient is in a clinical trial. Blue line and green line represent the disease stage before and after the application of the data management algorithm respectively. Brown line and pink line represent previous and following disease stage respectively (intermediate parameter implicated in data management rules). Purple line indicates whether the patient is part of a clinical trial. Black line indicates whether the diagnosis is defined. The bottom graphic presents the treatment line evolution. Blue line and green line represent the treatment line position before and after the application of the data management algorithm respectively.
(TIFF)

S2 Fig PRM centers location in France. This map shows the distribution of the 130 French medical centers participating in the PRM program as of 31 December 2019. Each center is represented by a dot on the map. When several centers are in the same city, the dots may overlap. The map also shows which part of the territory is covered by each of the six regions defined by the PRM program.
(TIF)

S1 Table List of Electronic Pharmacy Record (EPR) systems used by PRM centers. (DOCX)

S2 Table List of cancer drugs specific to the treatment of other cancer than BC or LC that result in the removal of the patient from the PRM database. (DOCX)

S3 Table Members of the independent PRM scientific committee. (DOCX)

S4 Table Description of the data management algorithms. (DOCX)

S5 TableResults of the quality control for each data management algorithms applied on random samples of breast and lung cancer patients.NA, Not Applicable. * Sample sizes were calculated using the following formula: $\frac{\frac{z^{2}\times p(1-p)}{e^{2}}}{1+(\frac{z^{2}\times p(1-p)}{e^{2}N})}$. e: the margin of error has been set at 10%. z: Z-score = 1,65. p: based on the most unfavorable hypothesis, that of a 50% estimation. ** Clopper-Pearson interval method was used to calculate the 95% binomial confidence intervals.(DOCX)

S6 Table Distributions of breast cancer and lung cancer patients therapeutic management, by medical center category and geographic location, in PRM centers and in all French medical centers, according to the French National Hospital database (PMSI) in 2018. GH/NFPH, general hospitals and non-for-profit hospitals; UH/CCC, university hospitals and comprehensive cancer centers.
(DOCX)
