# Supplementary figures and images for: Personalized Reimbursement Model (PRM) program: A real-world data platform of cancer drugs use to improve and personalize drug pricing and reimbursement in France
Source: PLoS One. 2022 Apr 19;17(4):e0267242. doi: 10.1371/journal.pone.0267242 (PMC9017943; doi:10.1371/journal.pone.0267242)

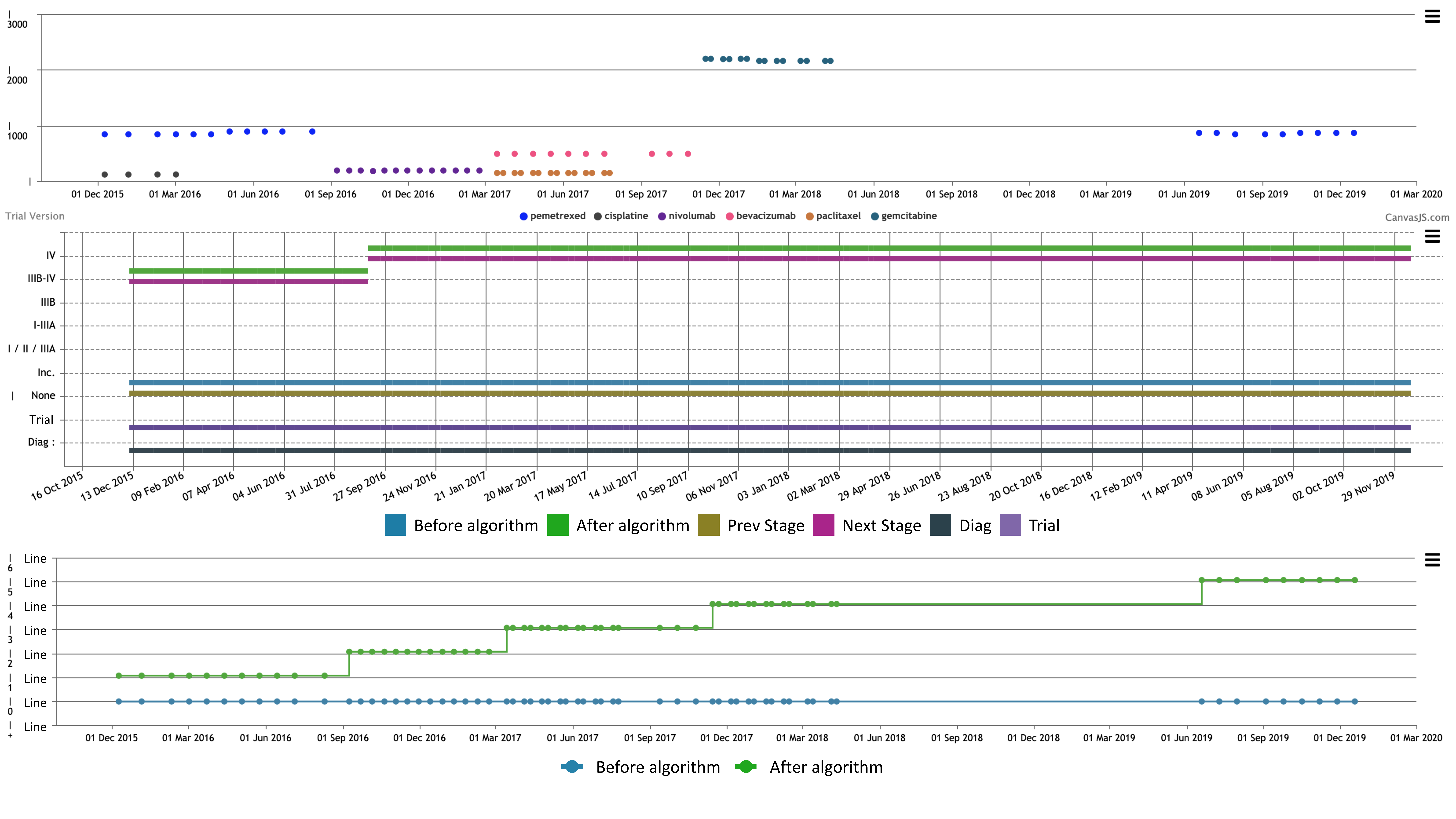

Supplement: S1 Fig — The user interface allows to show anonymized patient’s treatment history according to the treatment line and the disease stage. Patient ID, centre ID, age, gender, weight and height have been removed to ensure patient anonymization. This example shows the contribution of the data management algorithmto recover treatment lines and disease stage that were initially incomplete or missing, based on patient’s available data in the database. The top graphic presents the patient cancer drug history: the international nonproprietary name (INN) of the cancer drugs administered, the dose in mg and the date of administration for each cancer drugs administered. The graphic in the middle presents disease stage evolution and periods when the patient is in a clinical trial. Blue line and green line represent the disease stage before and after the application of the data management algorithm respectively. Brown line and pink line represent previous and following disease stage respectively (intermediate parameter implicated in data management rules). Purple line indicates whether the patient is part of a clinical trial. Black line indicates whether the diagnosis is defined. The bottom graphic presents the treatment line evolution. Blue line and green line represent the treatment line position before and after the application of the data management algorithm respectively. (TIFF) [file pone.0267242.s001.tiff]

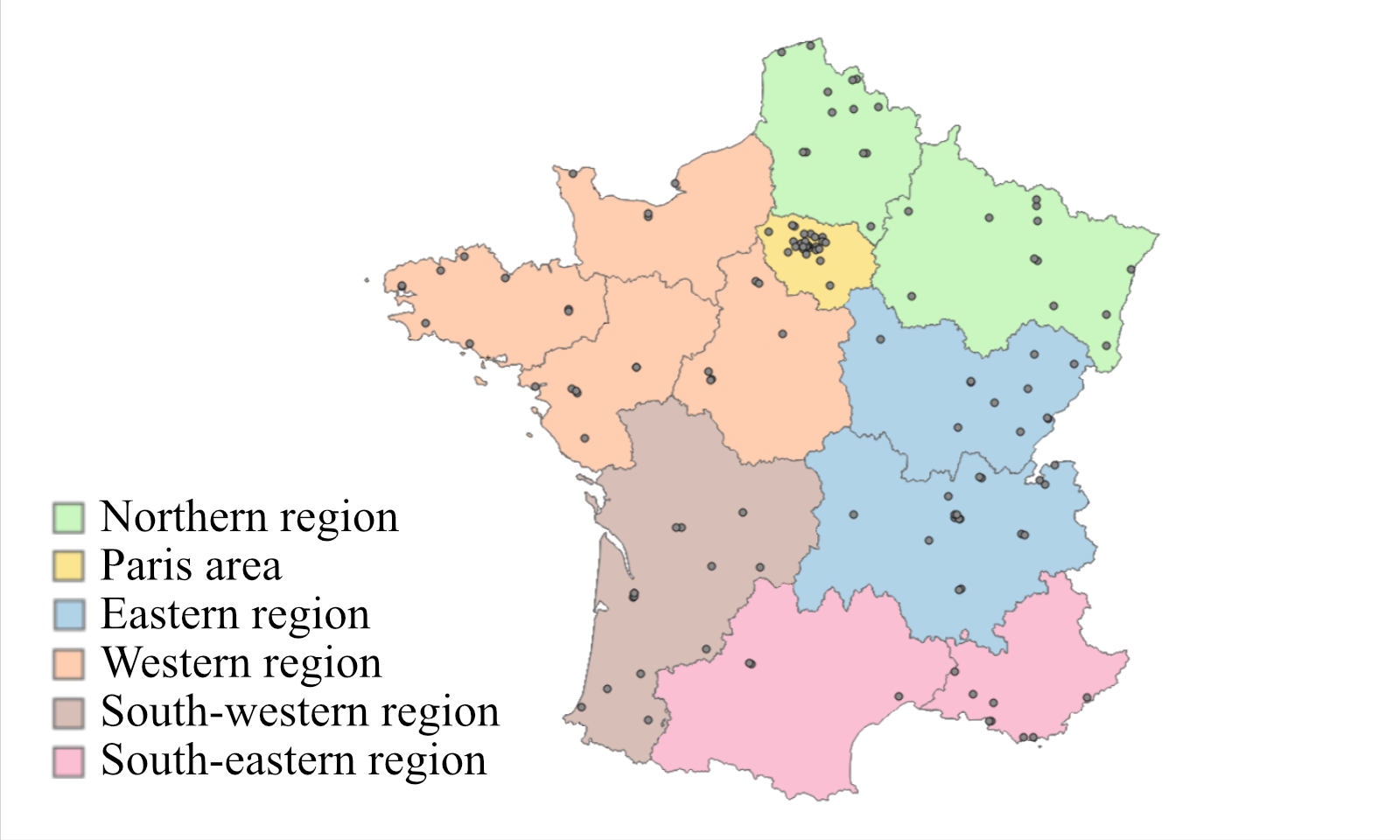

Supplement: S2 Fig — This map shows the distribution of the 130 French medical centers participating in the PRM program as of 31 December 2019. Each center is represented by a dot on the map. When several centers are in the same city, the dots may overlap. The map also shows which part of the territory is covered by each of the six regions defined by the PRM program. (TIF) [file pone.0267242.s002.tif]
