# Supplementary material for: Personalized Reimbursement Model (PRM) program: A real-world data platform of cancer drugs use to improve and personalize drug pricing and reimbursement in France
Source: PLoS One. 2022 Apr 19;17(4):e0267242. doi: 10.1371/journal.pone.0267242 (PMC9017943; doi:10.1371/journal.pone.0267242)
Supplement: S1 Table — (DOCX) [file pone.0267242.s003.docx]

**S1 Table**.

| **Electronic pharmacy record system names** | **Proportion of users** |
| --- | --- |
| CHIMIO | 78 % |
| TIMEWISE | 6% |
| ASCLEPIOS | 2% |
| ONCODOME | 1% |
| OncoBase | 2% |
| CristalNet | 1% |
| BPC | 6% |
| In-house developed EPR system | 4% |
