# Supplementary material for: Personalized Reimbursement Model (PRM) program: A real-world data platform of cancer drugs use to improve and personalize drug pricing and reimbursement in France
Source: PLoS One. 2022 Apr 19;17(4):e0267242. doi: 10.1371/journal.pone.0267242 (PMC9017943; doi:10.1371/journal.pone.0267242)
Supplement: S2 Table — (DOCX) [file pone.0267242.s004.docx]

S2 Table.

| **List for BC patients** | **List for LC patients** |
| --- | --- |
| folic acid | levofolinic acid |
| levofolinic acid | aflibercept |
| actinomycin d | azacitidine |
| amifostine | bendamustine |
| azacitidine | bortezomib |
| bendamustine | cetuximab |
| bleomycin | cytarabine |
| bortezomib | dacarbazine |
| cabazitaxel | daratumumab |
| cetuximab | irinotecan |
| cladribine | methotrexate |
| dacarbazine | mitomycin c |
| estramustine | oxaliplatin |
| fludarabine | trastuzumab |
| irinotecan | arsenic trioxide |
| mitomycin c | vinflunine |
| mitoxantrone |  |
| ofatumumab |  |
| panobinostat |  |
| pemetrexed |  |
| raltitrexed |  |
| ramucirumab |  |
| rituximab |  |
| sunitinib |  |
| temozolomide |  |
| trebaninib |  |
| arsenic trioxide |  |
| vinflunine |  |
