# Supplementary material for: Personalized Reimbursement Model (PRM) program: A real-world data platform of cancer drugs use to improve and personalize drug pricing and reimbursement in France
Source: PLoS One. 2022 Apr 19;17(4):e0267242. doi: 10.1371/journal.pone.0267242 (PMC9017943; doi:10.1371/journal.pone.0267242)
Supplement: S3 Table — (DOCX) [file pone.0267242.s005.docx]

S3 Table.

| **Name** | **Position** | **Medical center** | **Expertise** |
| --- | --- | --- | --- |
| Mr. Olivier Aujoulat | Medical director of pharmaceutical services division | Groupe hospitalier de la region de Mulhouse et Sud Alsace of Mulhouse | Expertise in pharmaceutical services division management |
| Prof. Christos Chouaid | Head of pneumology department | Centre Hospitalier Intercommunal, Créteil | *Expertise in lung cancer management, real world data and medico-economic analyses* |
| Dr. Bruno Coudert | Head of medical oncology department,  Medical advisor for IT systems | Centre de lutte contre le cancer Georges François Leclerc, Dijon | *Expertise in breast cancer and lung cancer management and hospital IT systems* |
| Dr. Didier Debieuvre | Head of pneumology department | Centre Hospitalier Emile Muller, Mulhouse, | *Expertise in lung cancer management and real world data* |
| Mrs. Anne Doly | Former director of IT division | Centre Jean Perrin, Clermont Ferrand | *Expertise in hospital IT systems and database management* |
| Prof. Nicolas Girard | Head of oncologic pneumology department | Institut Curie, Paris | *Expertise in lung cancer management and real world data* |
| Dr. Werner Hielgers | Medical coordinator of oncologic urology department | Institut Sainte Catherine, Avignon | *Expertise in lung cancer and urologic cancer management* |
| Mr. Julien Manson | Head of cancer drug departement | Centre Hospitalier René-Dubos, Pontoise | *Expertise in hospital pharmacy IT systems* |
| Dr. David Pérol | Director of clinical research | Centre de lutte contre le cancer Léon Bérard, Lyon, | *Expertise in methodology, public health and health economics* |
