# Supplementary material for: Personalized Reimbursement Model (PRM) program: A real-world data platform of cancer drugs use to improve and personalize drug pricing and reimbursement in France
Source: PLoS One. 2022 Apr 19;17(4):e0267242. doi: 10.1371/journal.pone.0267242 (PMC9017943; doi:10.1371/journal.pone.0267242)
Supplement: S4 Table — (DOCX) [file pone.0267242.s006.docx]

S4 Table.

| **Algorithm** | **Algorithm description for LC patient** | **Algorithm description for BC patient** |
| --- | --- | --- |
| 1 | Regroups disease stages within lines corresponding to one administration with multiple drugs. If available disease stages are different, disease stage for this administration is categorized as inconsistent.  Eg : Stage IV + missing => **Stage IV**; Stage IV + Stage II => **Inconsistent** | Regroups disease stages within lines corresponding to one administration with multiple drugs. If available disease stages are different, disease stage for this administration is categorized as inconsistent.  Eg : Stage IV + missing => **Stage IV**; Stage IV + Stage II => **Inconsistent** |
| 2 | Fills in the gaps if disease stage is missing or categorized as inconsistent and enclosed with two identical disease stages.  Eg : *Stage IV > missing > Stage IV* ***=>*** ***Stage IV*** | Fills in the gaps if disease stage is missing or categorized as inconsistent and enclosed with two identical disease stages.  Eg : *Stage IV > missing > Stage IV* ***=>*** ***Stage IV*** |
| 3 | Replaces missing stages and inconsistent stages if they are enclosed with two disease stages that correspond to early disease stages (stage I or II or IIIA or I-IIIA).  *Eg : Stage II > missing > Stage IIIA* ***=>*** *Stage II >* ***Stage I-IIIA*** *> Stage IIIA* | Replaces missing stages and inconsistent stages if they are enclosed with two disease stages that correspond to early disease stages (stage I or II or IIIA or I-IIIA).  *Eg : Stage II > missing > Stage IIIA* ***=>*** *Stage II >* ***Stage I-IIIA*** *> Stage IIIA* |
| 4 | Replaces missing stages and inconsistent stages if they are enclosed with two disease stages that correspond to metastatic disease stages (stage IIIB or IV or IIIB-IV).  *Eg : Stage IIIB > missing > Stage IV* ***=>*** *Stage IIIB >* ***Stage IIIB-IV*** *> Stage IV* | Replaces missing stages and inconsistent stages with Stage I-IIIA if they are followed by early disease stages (stage I or II or IIIA or I-IIIA) with the same treatment regimen when the delay between the start of the early stage and the beginning of the missing disease stage sequence is shorter than 4 months. |
| 5 | Replaces missing stages and inconsistent stages with Stage I-IIIA if they are followed by early disease stages (I ou II ou IIIA ou I-IIIA).  *Ex : missing > Stage IIIA* ***=>*** ***Stage I-IIIA*** *> Stage IIIA* | Replaces missing stages and inconsistent stages with Stage IIIB-IV if they are followed by a metastatic disease stage (IIIB or IV or IIIB-IV) with the same treatment regimen when the delay between the start of a metastatic disease stage and the beginning of the missing disease stage sequence is shorter than 4 months. |
| 6 | Replaces missing stages and inconsistent stages with Stage IIIB-IV if they are followed by metastatic disease stages (IIIB ou IV ou IIIB-IV) with the same treatment regimen when the delay between the start of a metastatic disease stage and the beginning of the missing disease stages is shorter than 4 months.  Eg : *missing (Pemetrex + Bevacizumab + Paclitaxel regimen) > Stage IV (Pemetrex + Bevacizumab + Paclitaxel regimen)* ***=>*** ***Stage IIIB-IV*** *> Stage IV* | Any disease stage following a metastatic disease stage (IIIB or IIIB-IV or IV) is replaced with a metastatic disease stage. |
| 7 | Any disease stage following a metastatic disease stage (IIIB or IIIB-IV or IV) is replaced with a metastatic disease stage. | NA |
| 8 | A ULTIMATE regimen with bevacizumab + taxane with paclitaxel forces disease stage to metastatic disease stage (IIIB or IIIB-IV or IV, if disease stage is available for this treatment line) and treatment line to 2 if no other treatment line was provided by the center. | A drug identified as late stage drug, forces disease stage to metastatic disease stage (IIIB or IIIB-IV or IV, if disease stage is available for this treatment line) |
| 9 | A regimen with bevacizumab + platinium + pemetrexed or bevacizumab + platinium + taxane forces disease stage to metastatic disease stage (IIIB or IIIB-IV or IV, if disease stage is available for this treatment line) and treatment line to 2 if no other treatment line was provided by the center. | A regimen with an intrathecal administration (identified from the name of the treatment regimen) forces disease stage to metastatic disease stage (IV) on the first date of administration of the regimen. |
| 10 | A regimen with immunotherapy such as *atezolizumab* ou *nivolumab* ou *durvalumab* ou *pembrolizumab* forces disease stage to metastatic disease stage (IIIB or IIIB-IV or IV, if disease stage is available for this treatment line) and treatment line to 2 when it corresponds to atezolizumab or nivolumab or to 1 when it corresponds to durvalumab or pembrolizumab and if no other treatment line was provided by the center. | If treatment response indicates “late stage”, forces disease stage to late stage (IIIB or IIIB-IV or IV) on the first date of the treatment response. |
| 11 | A regimen with an intrathecal administration (identified from the name of the treatment regimen) forces disease stage to metastatic disease stage (IV) on the first date of administration of the regimen. | A regimen with cyclophosphamid forces disease stage to early disease stage (stage I or II or IIIA or I-IIIA) |
| 12 | A physiologic indicator indicates “metastases = yes” forces disease stage to metastatic disease stage (IV) on the first date of the physiologic indicator. | Replaces any disease stage following a metastatic stage that was forced by ASR1 rule with late stage. |
| 13 | Any disease stage after a disease stage among IIIB, IIIB-IV and IV is replaced with IIIB-IV or IV. | NA |
| 14 | Any missing disease stage and inconsistent disease stage, after the application of all the above rules, is replaced with IIIB-IV. | NA |
