# Supplementary material for: Personalized Reimbursement Model (PRM) program: A real-world data platform of cancer drugs use to improve and personalize drug pricing and reimbursement in France
Source: PLoS One. 2022 Apr 19;17(4):e0267242. doi: 10.1371/journal.pone.0267242 (PMC9017943; doi:10.1371/journal.pone.0267242)
Supplement: S5 Table — NA, Not Applicable. * Sample sizes were calculated using the following formula: z2×p(1−p)e21+(z2×p(1−p)e2N). e: the margin of error has been set at 10%. z: Z-score = 1,65. p: based on the most unfavorable hypothesis, that of a 50% estimation. ** Clopper-Pearson interval method was used to calculate the 95% binomial confidence intervals. (DOCX) [file pone.0267242.s007.docx]

S5 Table.

| **Results of the quality control for breast cancer patient** | | | |
| --- | --- | --- | --- |
| Algorithm | random sample reviewed* (n) | Data management algorithm correctly applied (n) | Score % [95CI]** |
| 1 | 67 | 67 | 100% [95.6; 100] |
| 2 | 67 | 66 | 98,5% [92; 100] |
| 3 | 67 | 67 | 100% [95.6; 100] |
| 4 | 66 | 65 | 98,5% [91.8; 99.8] |
| 5 | 65 | 63 | 96,9% [89.4; 99.6] |
| 6 | 65 | 62 | 95,4% [87.1; 99] |
| 7 | NA | NA | NA |
| 8 | 68 | 68 | 100% [94.7; 100] |
| 9 | 28 | 28 | 100% [87.7; 100] |
| 10 | 63 | 63 | 100% [94.3; 100] |
| 11 | 68 | 64 | 94,1% [85.6; 98.4] |
| 12 | 67 | 67 | 100% [94.6; 100] |
| 13 | NA | NA | NA |
| 14 | NA | NA | NA |
| **Results of the quality control for lung cancer patient** | | | |
| Algorithm | random sample reviewed*(n) | Data management algorithm correctly applied (n) | Score % [95CI]** |
| 1 | 54 | 53 | 98,1% [90.1; 100] |
| 2 | 64 | 63 | 98,4% [91.6; 100] |
| 3 | 34 | 34 | 100% [89.7; 100] |
| 4 | 64 | 64 | 100% [94.4; 100] |
| 5 | 33 | 33 | 100% [89.4 100] |
| 6 | 63 | 63 | 100% [94.3; 100] |
| 7 | 63 | 63 | 100% [94.3; 100] |
| 8 | 64 | 64 | 100% [94.4; 100] |
| 9 | 66 | 66 | 100% [94.6; 100] |
| 10 | 65 | 65 | 100% [94.5; 100] |
| 11 | NA | NA | NA |
| 12 | 58 | 58 | 100% [93.8; 100] |
| 13 | 63 | 62 | 98,4% [91.5; 100] |
| 14 | 63 | 63 | 100% [94.3; 100] |
