# Supplementary material for: Personalized Reimbursement Model (PRM) program: A real-world data platform of cancer drugs use to improve and personalize drug pricing and reimbursement in France
Source: PLoS One. 2022 Apr 19;17(4):e0267242. doi: 10.1371/journal.pone.0267242 (PMC9017943; doi:10.1371/journal.pone.0267242)
Supplement: S6 Table — GH/NFPH, general hospitals and non-for-profit hospitals; UH/CCC, university hospitals and comprehensive cancer centers. (DOCX) [file pone.0267242.s008.docx]

S6 Table

|  | **Patients with breast cancer in PRM centers** | | | **Patients with brest cancer across the country** | | |
| --- | --- | --- | --- | --- | --- | --- |
|  | **n** | | **%** | **n** | | **%** |
| **Northern region** | **3,866** | **14%** | | **10,385** | **17%** | |
| GH/NFPH | 1,693 | 6% | | 3,256 | 5% | |
| UH/CCC | 214 | 1% | | 2,989 | 5% | |
| Private hospitals | 1,959 | 7% | | 4,140 | 7% | |
| **Eastern region** | **5,338** | **20%** | | **10,038** | **16%** | |
| GH/NFPH | 1,320 | 5% | | 3,785 | 6% | |
| UH/CCC | 2,065 | 8% | | 3,376 | 6% | |
| Private hospitals | 1,953 | 7% | | 2,877 | 5% | |
| **South-Eastern and South-Western regions** | **5,068** | **19%** | | **16,141** | **26%** | |
| GH/NFPH | 1,584 | 6% | | 5,120 | 8% | |
| UH/CCC | 1,288 | 5% | | 5,667 | 9% | |
| Private hospitals | 2,196 | 8% | | 5,354 | 9% | |
| **Western region** | **7,115** | **26%** | | **12,346** | **20%** | |
| GH/NFPH | 1,466 | 5% | | 3,427 | 6% | |
| UH/CCC | 3,639 | 13% | | 4,390 | 7% | |
| Private hospitals | 2,010 | 7% | | 4,529 | 7% | |
| **Paris area** | **5,596** | **21%** | | **12,039** | **20%** | |
| GH/NFPH | 995 | 4% | | 2,196 | 4% | |
| UH/CCC | 3,007 | 11% | | 6,707 | 11% | |
| Private hospitals | 1,594 | 6% | | 3,136 | 5% | |
| **Total** | **26,983** | **100%** | | **60,949** | **100%** | |
|  | **Patients with lung cancer in PRM centers** | | | **Patients with lung cancer in PRM centers** | | |
|  | **n** | **%** | | **n** | **%** | |
| **Northern region** | **3,234** | **19%** | | **8,545** | **17%** | |
| GH/NFPH | 1,859 | 11% | | 4,755 | 9% | |
| UH/CCC | 336 | 2% | | 1,968 | 4% | |
| Private hospitals | 1,039 | 6% | | 1,822 | 4% | |
| **Eastern region** | **2,815** | **17%** | | **8,630** | **17%** | |
| GH/NFPH | 1,228 | 7% | | 4,108 | 8% | |
| UH/CCC | 891 | 5% | | 3,235 | 6% | |
| Private hospitals | 696 | 4% | | 1,287 | 3% | |
| **South-Eastern and South-Western regions** | **3,893** | **23%** | | **15,260** | **30%** | |
| GH/NFPH | 1,750 | 10% | | 6,278 | 12% | |
| UH/CCC | 1,042 | 6% | | 4,319 | 8% | |
| Private hospitals | 1,101 | 6% | | 4,663 | 9% | |
| **Western region** | **4,254** | **25%** | | **10,124** | **20%** | |
| GH/NFPH | 1,722 | 10% | | 4,782 | 9% | |
| UH/CCC | 1,699 | 10% | | 3,127 | 6% | |
| Private hospitals | 833 | 5% | | 2,215 | 4% | |
| **Paris area** | **2,825** | **17%** | | **8,349** | **16%** | |
| GH/NFPH | 1,847 | 11% | | 3,518 | 7% | |
| UH/CCC | 518 | 3% | | 3,634 | 7% | |
| Private hospitals | 460 | 3% | | 1,197 | 2% | |
| **Total** | **17,021** | **100%** | | **50,908** | **100%** | |
